# Object Recognition Memory: Distinct Yet Complementary Roles of the Mouse CA1 and Perirhinal Cortex

**DOI:** 10.3389/fnmol.2020.527543

**Published:** 2020-10-22

**Authors:** David A. Cinalli Jr., Sarah J. Cohen, Kathleen Guthrie, Robert W. Stackman Jr.

**Affiliations:** ^1^Jupiter Life Science Initiative, Charles E. Schmidt College of Science, Florida Atlantic University, Jupiter, FL, United States; ^2^Department of Psychology, Charles E. Schmidt College of Science, Florida Atlantic University, Boca Raton, FL, United States; ^3^Center for Complex Systems and Brain Sciences, Florida Atlantic University, Boca Raton, FL, United States; ^4^Department of Biomedical Science, Charles E. Schmidt College of Medicine, Florida Atlantic University, Boca Raton, FL, United States; ^5^FAU Brain Institute, Florida Atlantic University, Jupiter, FL, United States

**Keywords:** muscimol, Arc, hippocampus, object recognition, qRT-PCR

## Abstract

While the essential contribution of the hippocampus to spatial memory is well established, object recognition memory has been traditionally attributed to the perirhinal cortex (PRh). However, the results of several studies indicate that under specific procedural conditions, temporary or permanent lesions of the hippocampus affect object memory processes as measured in the Spontaneous Object Recognition (SOR) task. The PRh and hippocampus are considered to contribute distinctly to object recognition memory based on memory strength. Allowing mice more, or less, exploration of novel objects during the encoding phase of the task (i.e., sample session), yields stronger, or weaker, object memory, respectively. The current studies employed temporary local inactivation and immunohistochemistry to determine the differential contributions of neuronal activity in PRh and the CA1 region of the hippocampus to strong and weak object memory. Temporary inactivation of the CA1 immediately after the SOR sample session impaired strong object memory but spared weak object memory; while temporary inactivation of PRh post-sample impaired weak object memory but spared strong object memory. Furthermore, mRNA transcription and *de novo* protein synthesis are required for the consolidation of episodic memory, and activation patterns of immediate early genes (IEGs), such as c-Fos and Arc, are linked to behaviorally triggered neuronal activation and synaptic plasticity. Analyses of c-Fos and Arc protein expression in PRh and CA1 neurons by immunohistochemistry, and of Arc mRNA by qPCR after distinct stages of SOR, provide additional support that strong object memory is dependent on CA1 neuronal activity, while weak object memory is dependent on PRh neuronal activity. Taken together, the results support the view that both PRh and CA1 are required for object memory under distinct conditions. Specifically, our results are consistent with a model that as the mouse begins to explore a novel object, information about it accumulates within PRh, and a weak memory of the object is encoded. If object exploration continues beyond some threshold, strong memory for the event of object exploration is encoded; the consolidation of which is CA1-dependent. These data serve to reconcile the dissension in the literature by demonstrating functional and complementary roles for CA1 and PRh neurons in rodent object memory.

## Introduction

Declarative, or explicit, memory is dependent on several brain structures within the mammalian medial temporal lobe (Eichenbaum, 2000; Squire et al., 2004). Unimodal sensory information is conveyed from the perirhinal cortex (PRh) to the hippocampus *via* the lateral entorhinal cortex (LEC), while contextual/spatial information is conveyed from the parahippocampal and postrhinal cortices to the hippocampus *via* the medial entorhinal cortex (MEC; Burwell and Amaral, 1998; Witter and Amaral, 2004). Lesions of the hippocampus impair rodent performance on spatial learning and memory tasks (Morris et al., 1982; Riedel et al., 1999); results that have established the functional significance of the hippocampal formation to spatial and temporal memory processes (O’Keefe, 1976; Eichenbaum et al., 1990; McNaughton et al., 2006; Eichenbaum, 2014). Lesions of the hippocampus also disrupt non-spatial memory as assessed by several tasks (Cave and Squire, 1991; Bunsey and Eichenbaum, 1996; Eichenbaum et al., 1999; Alvarez et al., 2001). However, non-spatial object memory has been attributed to the PRh based on studies of primates and rodents (Buffalo et al., 1999; Winters and Bussey, 2005). Indeed, compelling evidence demonstrates that lesions of the rodent PRh impair object recognition memory while sparing spatial memory (Winters et al., 2004). Such findings have promoted the view that while the PRh supports item (i.e., what) memory, the hippocampus supports spatial (i.e., where) memory. This dichotomous argument complicates our understanding of the neural mechanisms underlying event memory—that is, the memories for what has happened within the context of space and time. Interestingly, lesions of the PRh affect the location-specific firing properties of rat CA1 hippocampal neurons (Muir and Bilkey, 2001). Further, the functional inactivation of the PRh disrupts the performance of an object-place paired association task, likely by affecting object-place firing correlates of CA1 neurons in the hippocampus (Lee and Park, 2013). These results indicate that the PRh contributes significantly to hippocampal representations of spatial information and conjunctive representations of object-in-place. Finally, several reports indicate that under certain circumstances the rodent hippocampus is critical for object recognition memory (Cave and Squire, 1991; Wood et al., 1993; Clark et al., 2000; De Lima et al., 2006; Rossato et al., 2007; Broadbent et al., 2010; Clarke et al., 2010; Cohen et al., 2013; Stackman et al., 2016). Mixed results of the effects of PRh and hippocampal lesions on object memory indicate that there may be multiple, to be determined, principles by which these regions operate within memory circuits. Perhaps both structures are necessary for object memory but in different capacities, as we suggested in a recent review article (Cohen and Stackman, 2015). Additional systematic experiments are needed to reconcile the interaction of structures within the temporal lobe memory system.

The functional dichotomy between the hippocampus and the PRh has been heavily studied, resulting in different theories for the types of non-spatial memory processes supported by each region. One theory posits that recollection is attributed to the hippocampus while familiarity is attributed to the PRh. For example, results indicate that rats with hippocampal lesions exhibit impaired recollection but enhanced familiarity, arguing that these structures are either functionally independent with hippocampus mediating recollection only (Fortin et al., 2004; Sauvage et al., 2008), or that the two regions can interact competitively. Similarly, local silencing of rat PRh neuronal activity by microinfusion of lidocaine impairs object memory, a result interpreted as evidence that the PRh is necessary for familiarity (Winters and Bussey, 2005). An alternative view states that the functional difference between the hippocampus and PRh is concerning strong and weak forms of non-spatial object memory (Squire et al., 2007). According to this view, we assert that the functional difference between these structures is rooted in the gradient of object memory strength; strong object memory depends more on the hippocampus than PRh, and weak object memory depends more on PRh than the hippocampus. The apparent variability in the reported contributions of these structures to object memory may stem from differences in the experimental protocol used. To investigate this concept in mice, we manipulated the Spontaneous Object Recognition (SOR) sample session exploration criterion to produce either strong or weak memories. Preliminary studies demonstrated that limiting the duration of sample session object exploration to shorter or longer periods affected the strength of the resulting object memory as demonstrated by relative differences in object discrimination during the subsequent test session. Bilateral inactivation of CA1 of the dorsal hippocampus after weak object memory training did not affect test session discrimination performance; however, inactivation of CA1 after strong object memory training significantly impaired object discrimination. In contrast, bilateral inactivation of PRh after weak memory training significantly impaired object discrimination, but inactivation after strong object memory training did not affect object discrimination. These findings provide compelling evidence that the hippocampus supports strong object memory, while the PRh supports weak object memory.

Also, immunohistochemical techniques were employed to quantify the first proteins produced following neuronal activation, known as immediate early gene (IEG) proteins (Jones et al., 2001). Classically, neuronal expression of the IEGs, Fos, and the activity-regulated cytoskeletal protein (Arc), has been used to map rodent brain regions that are recruited at specific time points within a given task (Kubik et al., 2007; Kovács, 2008; Kawashima et al., 2014). We designed our IEG behavioral protocol similar to our inactivation studies to permit comparisons of c-Fos and Arc protein expression between CA1 and PRh neurons after strong and weak object memory training and testing conditions. Significant increases in IEG protein expression were only observed in CA1 neurons following a strong object memory sample session. However, a weak object memory sample session elicited increased IEG protein expression only in PRh neurons. To gain a better understanding of how Arc protein levels are modulated to support this double dissociation in the object memory circuitry, Arc mRNA expression was also quantified. These immunohistochemical results were largely supported by quantitative real-time PCR (qRT-PCR) analyses of Arc mRNA expression in hippocampal and PRh samples after weak and strong object training. Based on both our pharmacological inactivation and IEG findings, we assert that strong object memory relies on the CA1 region of the dorsal hippocampus and weak object memory on PRh. Taken together, the results of the present studies support the view that the functionally distinct contributions of these two medial temporal lobe structures to object memory is dependent on memory strength.

## Materials and Methods

### Mice

Male C57BL/6J mice (7–10 week old; Jackson Labs, Bar Harbor, ME) were housed 4 per cage with *ad libitum* access to food and water. The room temperature was maintained at 22 ± 4°C and humidity at 50 ± 5%. A 12-h light/dark cycle was maintained beginning at 7:00 AM. All experimental procedures were conducted during the light period following NIH guidelines; procedures were reviewed and approved by the Florida Atlantic University’s Institutional Animal Care and Use Committee before the initiation of experiments. For mice used in the inactivation experiments (*n* = 90), guide cannulae were implanted after 1-week acclimatization to the vivarium, and testing began 7 days post-operatively when the mice were 9 week old. For mice used in the immunostaining experiments (*n* = 66), testing began after 1-week acclimatization to the vivarium, when the mice were 8 week old.

### Intrahippocampal Cannulation and Microinfusion

For the inactivation experiments, mice were implanted with chronic bilateral guide cannulae (Plastics One, Inc., Roanoke, VA, USA) above the CA1 region of the dorsal hippocampus (A/P − 2.0 mm, M/L ± 1.5 mm, D/V − 1.1 mm from Bregma; corresponding to intermediate CA1), as previously described (Cohen et al., 2013), or above the PRh (A/P − 2.25 mm, M/L ± 4.0 mm, D/V − 3.25 mm from Bregma; Franklin and Paxinos, 2008). Behavioral testing began 7 days later to permit postoperative recovery. Each mouse received a “mock infusion” each day for 2 days, immediately after the arena habituation, to acclimate the mice to the microinfusion procedure, as previously described (Cohen et al., 2013). For the actual microinfusions, mice were briefly restrained while caps and dummy cannula were replaced with infusion cannula and received bilateral (0.35 μl/side, 0.33 μl/min) intra-CA1/intra-PRh muscimol (Tocris, 1 μg/μl in 0.9% sterile saline) or 0.9% sterile saline immediately after the sample session. For both mock and actual microinfusions, the mice were awake for the entire 3-min procedure; after placement of the infusion cannulae, the mice were released into an empty holding cage and allowed to freely explore. The procedures for the actual bilateral microinfusion followed that described above for the mock infusion; however, this time the inserted infusion cannulae penetrated 1 mm beyond the tip of the guide cannulae to achieve bilateral intra-CA1/intra-PRh infusion.

### Spontaneous Object Recognition (SOR) Tasks and Protocols for Inactivation Studies

**Figure 1:**
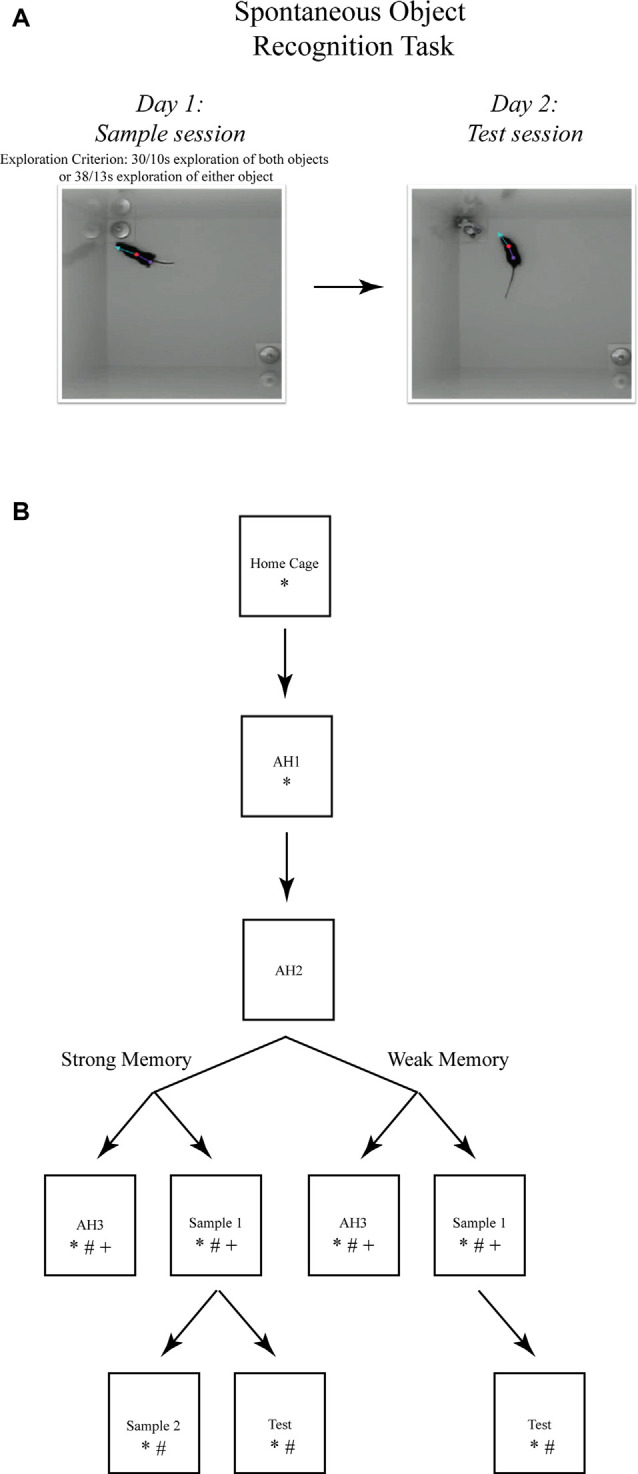
The spontaneous object recognition (SOR) task. **(A)** The object recognition memory task protocol consisted of a sample session (left) and a test session (right) conducted within a familiar rectangular arena. During the sample session, the mouse was placed into the familiar arena to freely explore two presented objects until a prescribed sample session exploration criterion was reached. The criteria for strong object memory training was 38 s of exploration of one sample session object, or 30 s of exploration of both objects. The criteria for weak object memory training was 13 s of exploration on one object or 10 s of exploration of both objects. After a 24 h delay, the mouse was returned to the familiar arena for a 5 min test session in which one of the sample objects was replaced with a novel object. Object memory was inferred from an analysis of the differences in time spent exploring both test session objects. These photographs depict an example of the arena configuration and the objects our lab has used to test object memory in mice using the SOR task (Cohen et al., 2013). **(B)** The SOR task protocols used for immunohistochemical staining of immediate early genes (IEGs) and quantification of mRNA after strong or weak object memory training. Each box represents a behavioral session and each arrow represents a 24 h delay between sessions. *, signifies a group of mice euthanized following that session for Fos protein quantification; ^#^, signifies a group of mice euthanized following that session for Arc protein quantification; and ^+^, signifies a group of mice euthanized following that session for *Arc* mRNA quantification. Boxes located next to one another, and connected by a common arrow, indicate that the groups were matched for the time in the testing arena.

For all experiments, the apparatus consisted of two open-top, high-walled square arenas made of white ABS (each: 37.5 × 37.5 × 50 cm). For the inactivation experiments, during days 1 and 2, each mouse was habituated to one of the arenas for a 10-min empty arena session. On days 3 and 4, each mouse received one sample session and one test session, respectively, in the habituated (i.e., familiar) arena. During the sample session, each mouse was returned to the familiar arena that now contained two identical novel 3D objects (stainless steel cabinet leveling feet, each attached to a Plexiglas base, 4.2 cm dia and 6.0 cm tall, or plastic toy gorillas, each attached to a Plexiglas base) positioned in the northwest and southeast corners. To test strong object memory, each mouse was removed from the arena upon accumulating 30 s of exploration of each object or 38 s of either object; a time limit of 10 min was set for this event to occur. To test weak object memory, each mouse was removed from the arena upon accumulating 10 s of exploration of each object or 13 s of either object; again, this event was required to occur within 10 min. Preliminary studies were performed in which different object exploration time criteria were imposed to determine memory strength at a 24-h delay. Mice that achieved the strong object memory exploration criteria (i.e., 30/38 s) exhibited an average 70% preference for the novel object during the test session imposed 24 h later, while mice that achieved the weak object memory exploration criteria (i.e., 10/13 s) exhibited an average 60% preference for the novel object during the test session 24 h later. Imposing the respective sample object exploration criteria had the added advantage of ensuring that all mice were matched for sample session performance. The data from five mice that failed to reach the sample session exploration criteria were removed from the analyses. Microinfusions were administered immediately after the mouse was removed from the arena. During the test session, presented 24 h later, the familiar arena contained one of the familiar objects and one novel object (see Figure 1A). The mouse was removed from the arena after 5 min. The objects, arena floor, and walls were cleaned with 10% ethanol after each session to remove olfactory cues.

A modified SOR protocol was used for the immunostaining and qRT-PCR experiments, to ensure that comparisons could be made between memory trained groups of mice and respective yoked control groups of mice (see Figure 1B). Groups of mice were perfused 90 min after each respective behavioral session. All behavioral testing data was digitally acquired by the EthoVision XT (Noldus Inc., Leesburg, VA, USA) software package. Object exploration was scored off-line from the digital video files by experimenters that were blind to the treatment condition of the mice. Object memory was inferred from the discrimination ratio—calculated for each mouse by subtracting the time spent exploring the familiar object from the time spent exploring the novel object, and then dividing the result by the total time spent exploring both items. Discrimination ratio scores range from −1 to 1, with positive scores indicating novel object preference, negative scores indicating familiar object preference, and a ratio = 0 indicating chance performance or a lack of preference for one object over another. Mice were randomly assigned to the different experimental conditions (i.e., weak memory or strong memory) ahead of any behavior evaluations to ensure unbiased group selections. However, it is important to note that for the IEG experiments, each mouse of the sample session groups was yoked to a mouse in the respective arena habituation session group mouse. In this manner, the actual time in the arena would not be a factor influencing IEG expression levels.

### Behavioral Data Analysis

Measures of latency (in seconds) to achieve the sample object exploration criterion were compared using two-tailed Student’s *t*-tests and discrimination ratio measures of saline- and muscimol-treated mice were compared using a three-factor ANOVA. For the immunostaining and qRT-PCR experiments, strong and weak object memory protocol groups were compared using two-tailed Student’s *t*-tests.

### Histology

**Figure 2:**
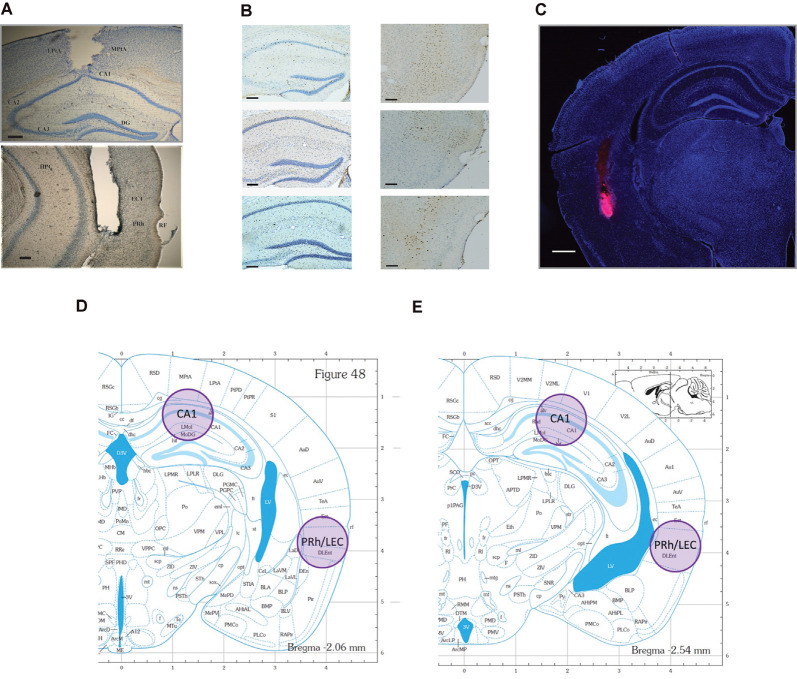
Representative photomicrographs of tissue sections analyzed for Fos and Arc protein expression and the representative locations where tissue punches were taken for analysis of *Arc* mRNA. locations. **(A)** CA1 (top), and PRh (bottom) photomicrographs of guide cannulae placement (scale bars: 200 μm). Data for mice with improper placement were removed from further analyses. **(B)** Representative photomicrographs of the regions where IEG proteins were counted. Neurons are stained blue, while IEG-positive protein is stained dark brown (scale bars: 100 μm). **(C)** Representative distribution of muscimol in perirhinal cortex (PRh). This figure depicts a representative example of drug distribution in mice that received bilateral infusions of fluorophore-conjugated muscimol (red fluorescence, BODIPY TMR-X, Molecular Probes). DAPI Fluoromount (Thermo-Fisher) was used to improve visualization of the locally infused fluorophore-conjugated muscimol within the tissue (scale bar: 500 μm). Analysis of images revealed that the fluorophore-conjugated muscimol diffused within the rhinal cortex, but largely remained within the PRh. For representative CA1 spread, see Cohen et al. (2013) and Stackman et al. (2016). **(D,E)** Shaded circles indicate tissue punch isolation regions for qRT-PCR against respective coronal plates from the mouse stereotaxic atlas (numbers refer to millimeter from Bregma; Franklin and Paxinos, 2008).

After the inactivation studies, each surgically implanted mouse was deeply anesthetized with 5% isoflurane and then the brain was dissected and preserved in 4% paraformaldehyde. Cannulae placements were confirmed by examination of cresyl violet-stained coronal 50 μm brain sections under a Nikon 55i light microscope. A subset of mice received a bilateral intra-PRh infusion of fluorophore-conjugated muscimol. Tissue was processed as above and then a Nikon 80i fluorescence microscope was used to image distribution within PRh. Data for any mice determined to have inappropriately placed cannulae were excluded from the analyses (*n* = 6). See Figures 2A,C for representative photomicrographs of appropriate cannula placement.

### Immunohistochemistry

Ninety min following the conclusion of testing, mice were deeply anesthetized with sodium pentobarbital (i.p.). Each mouse was transcardially perfused with 0.1 M phosphate buffer, followed by 4% paraformaldehyde, and brains were dissected and preserved in 4% paraformaldehyde for 5 days. Fixed brains were serially sectioned at 30 μm using a cryostat (Leica CM1850). Free-floating coronal sections were immunostained to localize Fos or Arc protein. Endogenous peroxidase activity was quenched by incubation in 1% hydrogen peroxide for 5 min. Sections were then blocked in 5% normal goat serum (NGS) and 0.3% Triton X-100 in 0.1 M phosphate buffer (PB) for 1 h. Tissue sections were then transferred to PB containing rabbit polyclonal antibody to Fos (Santa Cruz Biotech, sc-52) or Arc (R&D Systems, AF636) at 1:500 dilution, for incubation overnight at 20° C with rotation. Following rinses in PB, sections were incubated in biotinylated goat anti-rabbit secondary antibody (Thermo Fisher Scientific, NC9256157) at 1:200 dilution in PB with 3% NGS. After 2 h, the tissue was washed and treated with avidin-biotin-peroxidase enzyme complex (ABC, Vector Labs, PK-4000) at 1:40 dilution in PB for 90 min. Chromagen was developed with diaminobenzidine, and sections were then rinsed in PB, mounted on gelatin-coated microscope slides, counter-stained with Cresyl violet, and cover-slipped. Every third section was examined for immunostaining for Fos or Arc. To quantify Fos- or Arc-, positive neurons in the hippocampus and PRh, six images per mouse were taken at the septal, intermediate, and temporal levels of CA1. The cytoarchitectonic subregions were identified from coronal sections and regional borders correspond to the levels depicted in Figures 2D,E. Bilateral images were matched across all mice for each respective level. Representative images are presented in Figure 2B.

### Cell Counting

The tissue sections were analyzed using a brightfield Nikon Eclipse 55i compound microscope with a 10× objective, photographed using a Nikon DS-Fi1 camera (100× total magnification), and acquired using Nikon Elements software. Estimates of Fos- or Arc-positive cells in CA1 and PRh were made by both of the primary experimenters (DC and SC), who were blind to the experimental condition due to the randomization of the order the tissue sections were presented to confirm unbiased counting. Cytoarchitectonic subregions of CA1 of the dorsal hippocampus and rostral PRh were identified from coronal sections near AP −2.00 to −2.50 from Bregma, consistent with prior reports (Burwell, 2001; Bast et al., 2009). Preliminary staining was completed on tissue using a non-specific serum (secondary antibody only) to assess normal DAB background staining levels in the regions of interest. Clearly labeled cells in the target regions were counted if the color intensity of the respective stain was greater than that of the background. If the expression was ambiguous with the 10× objective, then localization was examined with a 40× objective for confirmation. Three bilateral sections were sampled from each mouse, such that analyzed sections were 90–150 μm apart (similar to the method described in Bernstein et al., 2019). The six total counts were averaged to generate the mean count for each mouse for each region. Group mean comparisons were made to test for significant differences in regional activation.

### Immunostaining Data Analysis

Averaged regional cell counts were normalized by dividing cell counts for each mouse by the average of the respective AH3 group (e.g., normalized count = total counts for a mouse that completed weak object training divided by the average number of counts for the weak AH3 group). This method of normalization was necessary to ensure that any activation differences between mice were not simply the result of time spent in the arena. Normalized counts were then analyzed using a three-factor (condition: AH3 vs. sample session; region: CA1 vs. PRh; memory strength: weak vs. strong) ANOVA, followed by Holm-Sidak multiple comparison tests where appropriate. Group comparisons between the home cage and AH1, AH1 and AH3, S1 and S2, and S and T, were analyzed by two-tailed Student’s *t*-tests.

### RNA Isolation and qRT-PCR

Forty min following the conclusion of testing, mice were euthanized by rapid decapitation, and brains were quickly extracted and sliced into 1-mm thick coronal sections. Brain tissue from two adjacent 1-mm thick sections was pooled to increase the likelihood of successful RNA isolation. Samples were taken bilaterally from CA1 in the dorsal hippocampus and from PRh/LEC with a tissue biopsy punch 1 mm in diameter (see Figures 2D,E) and immediately frozen on dry ice. Punch samples from two adjacent sections were pooled for RNA isolation using TRIzol reagent (Ambion, 15596018) and purified using the RNeasy Mini Kit (Qiagen, kit # 74104). DNA removal was performed by on-column deoxyribonuclease digestion for 15 min using the RNase-Free DNase Set (Qiagen, 79254). cDNA was prepared using the High Capacity cDNA Reverse Transcription Kit (Applied Biosystems, kit # 4368814). Probes used to amplify *Arc* and *GAPDH* mRNAs were obtained from ThermoFisher (TaqMan, 4331182). qRT-PCR was performed with the Bio-Rad CFX96 Real-Time PCR detection system using the following amplification parameters: 95°C for 10 min; 50 cycles of 95°C for 15 s, 60°C for 15 s, 72°C for 15 s; 95°C for 15 s, 60°C for 15 s; to generate dissociation curves for PCR products. Reactions were performed in triplicate for each tissue sample.

### qRT-PCR Data Analysis

*Arc* mRNA levels were normalized to *GAPDH* levels measured in parallel. The data were analyzed by comparing C(*t*) values obtained for each experimental condition (e.g., Weak AH3 vs. Weak Sample; Strong AH3 vs. Strong Sample) with the ΔΔC(*t*) method (Tsankova et al., 2006; Robison et al., 2014). Comparisons were made between cohorts of mice tested with strong and weak object memory experimental protocols groups using two-tailed Student’s *t*-tests.

## Results

### Inactivation Findings

Naïve mice received bilateral microinfusions of saline or muscimol into the CA1 or PRh immediately after completing a sample session in which the mice acquired weak or strong object memory training by exploring two identical novel objects for 10 s each, or 30 s each, respectively (see Figure 2A for representative placement of CA1 and PRh injection sites). Each mouse received a test session 24 h after the sample session. Results of these inactivation experiments were analyzed by a three-factor (region: CA1 vs. PRh; treatment: saline vs. muscimol; memory strength: weak vs. strong) ANOVA on the discrimination ratio measures. The three-factor ANOVA yielded a significant region × treatment × memory strength interaction (*F*_(1,89)_ = 9.56, *P* < 0.01), a significant treatment × memory strength interaction (*F*_(1,89)_ = 3.88, *P* = 0.05), but a non-significant region × memory strength interaction (*F*_(1,89)_ = 0.99, n.s.). As expected, the ANOVA yielded a significant main effect of treatment (*F*_(1,89)_ = 7.69, *P* = 0.01), indicating that the discrimination ratio scores were significantly greater for the saline-treated mice as compared to the muscimol-treated mice. The ANOVA also yielded a significant main effect of memory strength (*F*_(1,89)_ = 4.35, *P* = 0.04), with the follow-up *post hoc* test indicating that discrimination ratio scores were greater for mice given strong object memory training as opposed to weak object memory training. Last, the ANOVA yielded a non-significant main effect of region (*F*_(1,89)_ = 0.44, n.s.), and a non-significant region × treatment interaction (*F*_(1,89)_ = 0.14, n.s.). Given the significant three-way interaction, *post hoc* tests were conducted to focus on the specific main effects. The results of the *post hoc* tests are summarized below according to memory strength and region, and details are provided as to respective numbers of mice per treatment group, etc.

### Strong Object Memory

#### Post-training CA1 Inactivation

**Figure 3:**
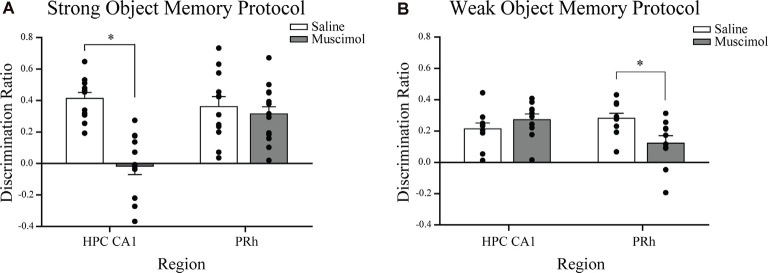
Influence of post-sample session local muscimol-induced inactivation of the CA1 or PRh on test session object discrimination depends on strength of object memory training. **(A)** Mice that achieved the strong object memory training criterion and then received an intra-CA1 infusion of muscimol exhibited significant impairment of object discrimination relative to the intra-CA1 saline-treated mice during the test session. Mice that achieved the strong object memory training criterion and then received an intra-PRh infusion of muscimol exhibited test session object discrimination that was comparable to that of intra-PRh saline-treated mice. **(B)** Mice that achieved the weak object memory training criterion and then received intra-CA1 muscimol exhibited object discrimination that was comparable to that of intra-CA1 saline-treated mice. Mice that achieved the weak object memory training criterion and then received intra-PRh muscimol exhibited significant impairment of object discrimination relative to the intra-PRh saline-treated mice. Note that the overall object discrimination performance of mice that received weak object memory training was significantly lower than that of mice that received strong object memory training. *P < 0.05 vs. vehicle group; mean ± SEM. Individual data points are represented by the small black filled circles superimposed over the respective group mean bar.

Naïve mice received bilateral intra-CA1 muscimol (*n* = 11) or saline (*n* = 12) immediately following the sample session. The sample session concluded for each mouse when the strong sample session exploration criterion event was completed within 10 min (30 s of exploration of each object or 38 s of either object). Both groups reached this sample session exploration criterion at similar times (saline 469 s, muscimol 459 s; *t*_(21)_ = 1.93, n.s.). During the test session, 24 h later, both groups spent equivalent amounts of time exploring the test session objects, but the mean discrimination ratio of the intra-CA1 muscimol group was significantly lower than that of the saline group [*t*_(21)_ = 5.93, *P* < 0.001, see Figure 3A, reprinted from Cohen et al. (2013)]. These findings suggest that inactivation of the CA1 region of the hippocampus after a sample session requiring considerable exploration of objects within a familiar context, prevented the consolidation of a strong object, or event memory (Cohen et al., 2013).

#### Post-training Perirhinal Cortex Inactivation

Naïve mice received bilateral intra-PRh muscimol (*n* = 15) or saline (*n* = 12) immediately following the mice achieving the strong sample session exploration criterion event (see Figure 2C for representative distribution of fluorophore-conjugated muscimol within PRh). Both groups reached the sample session exploration criterion at similar times (saline 565 s, muscimol 523 s; *t*_(25)_ = 1.30, n.s.). During the test session, 24 h later, both groups spent equivalent amounts of time exploring the test session objects and demonstrated similar discrimination of the novel object from that of the familiar (*t*_(25)_ = 0.62, n.s., see Figure 3A). These results suggest that inactivation of the PRh after a sample session requiring considerable exploration of the objects did not impair the consolidation of a strong object or event memory.

### Weak Object Memory

#### Post-training CA1 Inactivation

Naïve mice received bilateral intra-CA1 muscimol (*n* = 10) or saline (*n* = 10) immediately following the sample session. The sample session concluded for each mouse when the weak sample session exploration criterion was completed within 10 min (10 s of exploration of each object or 13 s of either object). Both groups reached sample session exploration criterion at similar times (saline 208 s, muscimol 153 s; *t*_(18)_ = 0.05, n.s.). During the test session, 24 h later, both groups spent equivalent amounts of time exploring the test session objects, and the mean discrimination ratios were also equivalent (*t*_(18)_ = −1.11, n.s., see Figure 3B). These findings suggest that inactivation of the hippocampus after a sample session requiring minimal exploration of the objects did not impair the consolidation of weak object memory.

#### Post-training Perirhinal Cortex Inactivation

Naïve mice received bilateral intra-PRh muscimol (*n* = 10) or saline (*n* = 10) immediately following the mice achieving the weak sample session exploration criterion. Both groups reached sample session exploration criterion at similar times (saline 184 s, muscimol 182 s; *t*_(18)_ = 0.07, n.s.). During the test session, 24 h later, both groups spent equivalent amounts of time exploring the test session objects; however, the mean discrimination ratio of the saline-treated mice was significantly greater than that of the muscimol-treated mice (*t*_(18)_ = 2.73, *P* = 0.01, see Figure 3B). These findings suggest that inactivation of the PRh after a sample session requiring minimal exploration of the objects significantly impaired the consolidation of weak object memory.

### Immunohistochemical Findings

#### Test Session Object Discrimination

**Figure 4:**
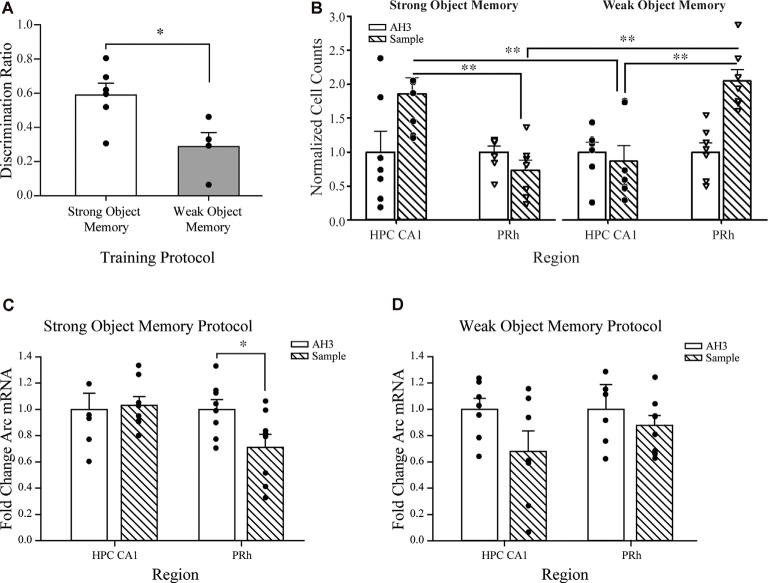
Object discrimination, Arc protein expression, and *Arc* mRNA quantification in mice that received strong or weak object memory training during a sample session of the SOR task. **(A)** Test session discrimination ratios of mice 24 h after training in the strong memory protocol are significantly greater than those of mice trained in the weak memory protocol. Importantly, both groups perform significantly above a chance ratio of 0. *P < 0.05. **(B)** Normalized counts of Arc-positive neurons in the CA1 region of the hippocampus and the PRh after completion of strong or weak object training or the respective arena habituation (AH3) session (see Figure 1B for a schematic diagram of the protocols). The graph illustrates the significant results from Holm-Sidak multiple comparison tests that followed the significant condition × region × memory strength interaction revealed by the three-factor ANOVA on normalized Arc counts (see “Materials and Methods” section “Spontaneous Object Recognition (SOR) Tasks and Protocols for Inactivation Studies” for complete details). Arc counts were significantly higher in the CA1 of mice that received strong object memory training compared to those in the PRh, as well as those of mice that received the strong AH3 session. Arc counts were significantly higher in the PRh of mice that received weak object memory training compared to those in the CA1, as well as those mice that received the weak AH3 session. **P < 0.001 vs. the respective region or memory strength as indicated by the overlying brackets. **(C)** A separate cohort of mice was used to examine *Arc* mRNA expression in the CA1 and PRh after strong or weak object training during a sample session or the respective strong or weak AH3 session. *Arc* mRNA expression in the CA1 was comparable between groups of mice that received strong object memory training and those that received AH3. However, *Arc* mRNA expression in the PRh/LEC was significantly less in mice that received strong object memory training than for mice that received the strong AH3 control session. *P < 0.05 vs. AH3 group. **(D)** For the weak object memory protocol, *Arc* mRNA expression in CA1 and in the PRh/LEC was not significantly different between mice that received weak object memory training compared to those that received the respective AH3 session. Data points and error bars on all graphs represent mean ± SEM. Individual data points are depicted in the respective panels by black filled circles and open triangles. Individual data points are represented by the small black filled circles, or open, inverted triangles, superimposed over the respective group mean bar.

Mean discrimination ratio scores were calculated for those mice that received a test session 24 h after the strong (*n* = 6) or weak object memory (*n* = 4) training session to confirm that the object memory inferred from the object discrimination was equivalent to that observed in the functional inactivation experiments. Mean discrimination ratios of both groups were significantly greater than chance (i.e., 0; strong memory: *t*_(5)_ = 8.53, *P* < 0.01; weak memory: *t*_(3)_ = 6.71, *P* < 0.01); however, the discrimination ratio of the strong object memory group was significantly greater than that of the weak object memory group (*t*_(8)_ = 2.76, *P* = 0.03, see Figure 4A).

#### Fos Protein Expression After Strong Object Training

The expression of Fos protein was quantified within the CA1 region of the hippocampus as a preliminary test of object memory-triggered activation during distinct stages of the SOR task. Planned comparisons were made of the Fos expression patterns between specific groups of mice (and given the preliminary nature of this experiment, we restricted quantification of Fos to only CA1 following strong object memory training). Mice (*n* = 8) were euthanized immediately upon removal from the home cage to obtain a baseline level of Fos expression in CA1. Comparisons were made to mice (*n* = 6) euthanized 90 min after an Arena Habituation 1 session (AH1). As predicted, exploration of a novel environment activated the dorsal hippocampus, and Fos expression in CA1 neurons was significantly increased compared to that of home cage mice (*t*_(12)_ = −7.15, *P* < 0.01). Counts of Fos-positive CA1 neurons in mice after AH1 (*n* = 6), were compared to those of mice sacrificed 90 min after AH3 (*n* = 7), revealing that numbers of Fos-positive neurons in CA1 were significantly greater for the AH1 mice compared to the AH3 mice (*t*_(11)_ = 3.47, *P* < 0.01). Presumably, this result was due to the higher environmental novelty associated with exploration during AH1 than that during AH3. Finally, to investigate CA1 neuronal activation in the presence of only one novel object, normalized counts of Fos-positive neurons for mice that experienced a second sample session (that is, exploration of two now familiar objects, S2; *n* = 8) were compared those of mice that experienced a test session (one familiar and one novel object, *n* = 8). As expected, the mean numbers of Fos-positive cells in CA1 was significantly greater for test session mice compared to S2 mice (*t*_(14)_ = −3.72, *P* < 0.01); likely reflecting activity related to the process of detecting the novel object and encoding a memory of it during the test session. These Fos imaging studies provided initial confirmation of the engagement of CA1 neurons in strong object memory. Next, we conducted a comparative analysis of Arc protein expression in CA1 and PRh neurons after weak or strong memory training, as Arc protein is a direct indicator of synaptic plasticity, interacting with cytoskeletal proteins (Plath et al., 2006; Korb et al., 2013).

#### Arc Protein Expression After Weak or Strong Object Training

As a more selective indicator of behaviorally triggered neuronal activity required for memory formation, we quantified numbers of neurons expressing Arc protein, known to play a crucial role in synaptic plasticity (Bramham et al., 2010; Gallo et al., 2018). Cell counts of CA1 and PRh Arc-positive neurons were made in cohorts of mice following the same behavioral protocol used to assess Fos expression described above, and in cohorts of mice following a weak object memory training session (see section “Behavioral Data Analysis”). To validate the accuracy of the staining technique used, groups of mice, with predictable activation patterns, were analyzed for Arc-positive neurons precisely as described for Fos (see section “Intrahippocampal Cannulation and Microinfusion”). Mice (*n* = 7) were sacrificed upon removal from the home cage to obtain an estimate of the baseline Arc expression in CA1 neurons. Comparisons were made to Arc-positive cell counts in CA1 from mice euthanized 90 min after an Arena Habituation 1 session (AH1; *n* = 7). Mean Arc-positive cell counts for AH1 mice were significantly greater than that of home cage mice (*t*_(12)_ = −5.50, *P* < 0.01); a predictable result. This finding confirmed that the staining protocol yielded appropriate patterns of activity-dependent Arc expression. As outlined in Figure 1, mice completed either a weak training or a strong training sample session. Respective groups of mice completed a third arena habituation session (yoked weak AH3 group or yoked strong AH3 group). Mice were euthanized and brains dissected 90 min after training. The mean distance traveled by the mice in the AH3 group (2,665.63 cm) was not significantly different than that of the respective sample session group (2,928.59 cm; *t*_(14)_ = 0.60, n.s.), suggesting that the presence of novel objects in the arena did not alter the perception of the context as being familiar. The novel, but otherwise non-threatening contexts tend to elicit greater amounts of exploratory locomotor responses in rodents. A subset of mice that received weak and strong object training was presented with a test session 24 h later. These mice were euthanized, and brains dissected 90 min after testing. Three tissue sections from the CA1 and PRh of each mouse were processed immunohistochemically to detect the neuronal expression of Arc protein.

First, to examine whether there were differences in Arc counts between the AH3 conditions, a two-factor (region: CA1 vs. PRh; “memory” strength: weak vs. strong) ANOVA was conducted on the raw Arc counts from mice that completed either the weak or strong AH3 control condition. The analysis revealed a significant strength × region interaction, *F*_(1,12)_ = 10.28; *P* < 0.01; a significant main effect of strength, *F*_(1,12)_ = 23.33, *P* < 0.001, and a significant main effect of region, *F*_(1,12)_ = 39.48; *P* < 0.001. The *post hoc* Holm Sidak tests following the significant interaction indicated a significant difference in raw Arc counts within the PRh between mice that completed a weak AH3 condition and those that completed a strong AH3 condition (*P* < 0.001). The raw PRh Arc counts following weak AH3 were higher than the PRh Arc counts following strong AH3. However, there was no significant difference in raw Arc counts within the CA1 between mice that completed a weak AH3 and those that completed a strong AH3 condition. *Post hoc* tests also yielded a significant difference in raw Arc counts between the PRh and CA1 for mice that completed the weak AH3 condition (*P* < 0.001), but no difference in raw Arc counts between the two regions for mice that completed the strong AH3 condition. These results suggest that exploration of the empty arena for the amount of time required of the weak AH3 condition and that for the strong AH3 condition did not differentially activate neurons of the CA1 region but did so for neurons of the PRh. The differences observed in raw Arc counts in the PRh after the two AH3 control conditions perhaps provide some degree of justification for our choosing to normalize Arc counts by the respective AH3 condition before pursuing analyses of Arc protein expression after the two object training conditions.

Next, a three-factor (condition: AH3 vs. sample session; region: CA1 vs. PRh; memory strength: weak vs. strong) ANOVA was conducted on the normalized Arc counts (see Figure 4). The analysis yielded a significant condition × region × strength interaction (*F*_(1,48)_ = 21.65, *P* < 0.001), indicating that the level of condition determined the effect of the region × strength interaction. Analysis of the three-way interaction revealed that the region × strength interaction was significant at the sample session level of the condition variable (*P* < 0.001), but not significant at the AH3 level (*P* = 0.568). The three-factor ANOVA also yielded a significant region × strength interaction (*F*_(1,48)_ = 14.75, *P* < 0.001), and a significant main effect of condition (*F*_(1,48)_ = 6.59, *P* < 0.015). Holm-Sidak pairwise multiple comparison tests revealed that Arc counts in the CA1 region were significantly higher after strong object training compared to after weak training (*P* < 0.001). This result suggests that some threshold duration of object exploration must be reached to up-regulate Arc protein expression in the CA1 region; 10 s of exploration of each sample object is not sufficient, while 30 s of exploration of each object is sufficient.

Arc counts were significantly higher in the PRh after weak object training compared to after strong training (*P* < 0.001). Arc counts were significantly higher in the CA1 than in the PRh after strong training compared to after weak training (*P* < 0.001), while Arc counts were significantly higher in the PRh after weak training compared to after strong training (*P* < 0.001). These Holm-Sidak test results are indicated by the asterisks in Figure 4B. This pattern of results is consistent with the interpretation of our inactivation data; that is, the consolidation of a strong object or event memory is not dependent upon neuronal activity in the PRh nor does this behavioral experience appear to drive synaptic plasticity there. The finding that the limited amount of exploration of, or exposure to, novel objects or stimuli dictated by the weak training protocol, elicited greater numbers of Arc-positive cells in the PRh as compared to CA1 of the hippocampus, is consistent with results of prior analyses of Fos expression (Wan et al., 1999, 2001; Barbosa et al., 2013), and was expected since object recognition memory processes have been widely attributed to the PRh.

Finally, the analyses revealed that Arc counts in CA1 after strong, but not weak, training was significantly greater than that after the respective AH3 session (*P* < 0.001). This pattern of results supports the view that neuronal activity and synaptic plasticity in CA1 is necessary for the consolidation of a strong object, or event memory. The data are also consistent with the view that the consolidation of a weak memory for objects explored does not engage CA1 neuronal activity or drive synaptic plasticity there. Likewise, Arc counts in PRh after weak, but not strong, training was significantly greater than that after the respective AH3 session (*P* < 0.001). For clarity, these significant results were not labeled in Figure 4B but will be fairly obvious to the reader. The differential impact of object training compared to an equivalently timed arena habituation session on the number of Arc expressing neurons in the CA1 and PRh runs counter to the argument that differences in Arc protein expression between strong and weak sample sessions are attributable to the time spent within the arena.

We conducted linear regression analyses to further examine the relationship between object training-induced Arc protein expression in the PRh and CA1 after a weak or strong sample session. The number of PRh neurons expressing Arc protein correlated with the number of CA1 hippocampal neurons expressing Arc protein in mice that had received weak object training; Pearson’s *r* = 0.91, *P* = 0.004. However, no correlation was found between the number of Arc protein-expressing neurons in the PRh and that in the CA1 in mice that had received strong object training, Pearson’s *r* = 0.25, n.s. While the significant correlation between regions after weak training is interesting and consistent with the results from the three-factor ANOVA described above, it should be considered with the proviso that caution should be exercised when interpreting results of regression analyses based on fewer than 10 pairs of observations.

Similar to the analysis of Fos immunolabeling reported in sections “Intrahippocampal Cannulation and Microinfusion,” a cohort of mice received strong object training during a sample session and then 24 h later, received either a test session or a second sample session (S2) as a control condition. Mice were euthanized and brains dissected 90 min after testing, and tissue sections from the CA1 were processed for Arc protein expression. Raw counts of Arc positive cells in CA1 were normalized against the average Arc cell counts from the S2 control group. Arc-protein expression in CA1 neurons was significantly higher in mice that had experienced the test session compared to the S2 group (Mean ± SEM, Test = 2.49 ± 0.58; S2 = 1.00 ± 0.19; *t*_(14)_ = −2.44, *P* < 0.05). The difference in CA1 Arc protein expression observed in the test session mice compared to that of the S2 mice likely reflects the process of discriminating the presented test session objects (one being novel) with the retrieved memory of the objects explored during the previous sample session. Except for the novel object, all other stimuli present during the test session are identical to that of the S2 condition. Presumably, both the test session and the S2 session experiences promote retrieval of the memory for the previous sample session, yet the respective groups of mice use that information distinctly. Thus, the significant difference in Arc protein expression in CA1 between the two groups of mice indicates the sensitivity of immunohistochemical analysis of activity-dependent Arc expression and provides further support for a critical role of CA1 in strong object memory.

### qRT-PCR Findings

#### Strong Object Memory

Arc mRNA expression was quantified within CA1 of the dorsal hippocampus and in PRh/LEC after strong object training during a sample session or after equivalently timed arena habituation sessions to further elucidate the molecular mechanisms underlying the induction of synaptic plasticity during object memory consolidation. The behavioral protocol was consistent with the Arc immunohistochemistry experimental design and planned comparisons analyses were conducted on *Arc* mRNA transcript expression following the exploration of objects to achieve the strong object memory training criterion (*n* = 8) or after exploration during the yoked AH3 session (*n* = 7). There was no significant difference in *Arc* mRNA expression in CA1 between the strong sample session and the AH3 session groups (*t*_(13)_ = −0.23; n.s., see Figure 4C), a result that is inconsistent with the increase in CA1 Arc protein expression observed in similarly trained groups of mice compared to AH3 mice. Thus, the increased expression of Arc protein in CA1 neurons after strong object memory training occurs independently of a net change in the level of *Arc* mRNA transcripts.

*Arc* mRNA was similarly measured in PRh/LEC samples after mice had achieved the strong sample exploration criterion (*n* = 8) or had received an AH3 session (*n* = 8). There was a significant decrease in *Arc* mRNA levels in PRh/LEC for mice that completed the strong sample session compared to those of the strong AH3 exploration groups (*t*_(14)_ = 2.34, *P* < 0.05, see Figure 4C). This result suggests that exploration of objects that reaches or exceeds our strong sample session criterion of 30-s on each object, or 38 s on one object triggers a down-regulation of *Arc* mRNA in PRh/LEC, which may account for the observed lack of significant increase in Arc-positive neurons in PRh following a strong object memory sample session. This pattern of results suggests a potential mechanism that down-regulates *Arc* mRNA transcripts in PRh/LEC; a mechanism that may be essential to the recruitment of CA1 during the consolidation of strong object memory. Such mechanisms for Arc mRNA degradation have been discussed (for reviews see Bramham et al., 2008, 2010).

#### Weak Object Memory

Arc mRNA expression was quantified within CA1 of the dorsal hippocampus and in PRh/LEC after weak object training during a sample session or after an equivalently timed arena habituation session. The behavioral protocol was consistent with the Arc immunohistochemistry experimental design, and planned comparisons were conducted on *Arc* mRNA transcripts of mice following exploration of objects to achieve the weak object memory training criterion (*n* = 7) or after exploration during the yoked AH3 session (*n* = 7). Analyses revealed no significant difference in *Arc* mRNA expression within CA1 in mice that had completed the weak sample session or a weak AH3 session (*t*_(12)_ = 1.82; n.s., see Figure 4D). This result is consistent with our immunohistochemistry results, described above, suggesting that *Arc* mRNA expression in CA1 was not affected by weak sample exploration, likely due to the lack of change in protein expression in CA1 following weak sample session training—given that CA1 neurons were not activated by this behavioral event. This result supports the view that CA1 activity is not critically involved in the consolidation of weak object memory.

A similar analysis of Arc mRNA expression in PRh/LEC in mice that had completed the weak sample session (*n* = 8) or a weak AH3 session (*n* = 7) revealed no significant difference between training groups (*t*_(13)_ = 0.64; n.s., see Figure 4D). The equivalent *Arc* mRNA expression levels between weak sample sessions and AH3 trained mice is in contrast to the observed significant increase in Arc protein expression in PRh neurons between similarly trained groups of mice. The increased Arc protein expression in the PRh after weak sample session training is independent of a net change in the level of *Arc* mRNA transcript expression, suggesting that weak training drives an increase in translation of *Arc* mRNA transcripts.

## Discussion

The present set of experiments was designed to investigate the differential contributions of the CA1 region of the hippocampus, and PRh to object memory in male C57BL/6J mice. Our experiments used local microinfusion of muscimol to temporarily block neuronal activity in the CA1 and PRh during object memory consolidation, and immunohistochemistry to stain for Fos and Arc proteins, and qRT-PCR to quantify Arc mRNA expression triggered by behaviors associated with the encoding, consolidation, and retrieval of object memory. It was predicted that if the amount of time the mice were permitted to explore objects during the sample session of the SOR task was increased or decreased, then the strength of the resulting memory of the objects would be altered accordingly. Based on test session measures of discrimination ratio, we characterized the resulting memory after a limited 10 s exploration of each sample session object (or 13 s of one object) as a weak object memory, while that after a more extensive 30 s exploration of each sample session object (or 38 s of one object) as a strong object memory. Indeed, the novel object preference exhibited by mice during the test session was significantly greater in mice that had completed 30/38 s exploration of the sample session objects (strong object memory training) compared to those mice that had only completed 10/13 s of exploration (strong object memory training). These two different sample session object exploration criteria were then used to test whether the formation of strong object memory or weak object memory differentially recruited CA1 or PRh neuronal activity.

Our experiments yielded several key results. First, the bilateral inactivation of CA1 after strong, but not weak, object memory training impaired object discrimination during the test session presented 24 h later. This result suggests that CA1 neuronal activity is essential for the consolidation of strong object memory or the memory of the event of exploring novel objects within a familiar context. The second key result was the complement: the bilateral inactivation of neuronal activity in the PRh after weak, but not strong, object memory training impaired object discrimination during the test session 24 h later. This result supports the view that PRh neuronal activity is required for the consolidation of object memory that guides discrimination based on object familiarity but is not necessary for the consolidation of stronger event memory. Although the local microinjections of muscimol were bilateral and restricted to the respective regions, both regions extend in the septo-temporal or rostral-caudal plane. Therefore, one might caution against concluding the role of a given hippocampal or extra-hippocampal structure based on the behavioral results after functional inactivation of a restricted subset of its neurons. For the present study, all aspects of the injection volume, rate, and respective cannula placements were consistent across the groups of mice. However, whether the bilateral injections of muscimol into the CA1 or PRh impaired test session behavior depended on the amount of time the mouse was permitted to explore objects during the sample session. Specifically, the same degree of post-training inactivation of neuronal activity in the PRh (e.g.,) only impaired novel object preference in those mice given weak memory training. Thus, the effect of inactivation appears to have been dependent on the degree of behavioral experience rather than any methodological difference. It is also important to note that by controlling the duration of the sample session by requiring the mice to accumulate 10 s or 30 s of sample object exploration, effectively matches all mice for sample session experience. This procedural control provides some assurance that the differences observed in subsequent test session behavior are most likely a consequence of the post-sample manipulation rather than due to sensorimotor, motivational, or attentional differences in the mice during the sample session.

In parallel with the regional inactivation experiments, we analyzed brain sections acquired from an additional cohort of mice to examine the behaviorally triggered expression of Arc protein, which is an IEG marker for neuronal plasticity. The Arc protein analyses revealed that a significantly larger ensemble of CA1 neurons was recruited as a result of the exploration of objects during the sample session of the strong memory protocol, as compared to the ensemble recruited as a result of the exploration of an empty arena during AH3. The increase in Arc protein-labeled neurons triggered by object exploration likely reflects the encoding and/or consolidation of strong object memory within CA1 and is similar to an earlier comparative study of c-Fos expression in CA1 and PRh after object exploration (Albasser et al., 2010). This finding, that strong object memory is supported by the activity of CA1 neurons, but not PRh neurons is consistent with the finding that the firing of individual PRh neurons is modulated by object familiarity (Ahn and Lee, 2015; Brown and Banks, 2015). The contribution of CA1 to strong object memory reported here is also consistent with previous reports that when mice are allowed to explore each sample object for at least 30 s, the memory that is encoded for that object is dependent on an intact and fully functioning hippocampus (Cohen et al., 2013; Tuscher et al., 2018). In contrast, analyses of Arc protein revealed that an equivalent, but lower number of CA1 neurons were recruited during the sample session of the weak object memory protocol and AH3. Moreover, the Arc protein analyses revealed that the CA1 neuronal ensemble recruited by the AH3 group yoked to the strong object memory trained group was not significantly different from that of the AH3 group yoked to the weak object memory trained group. This means that exploration of the empty familiar arena, regardless of time spent within the arena, did not differentially activate CA1 neurons. Therefore, we can conclude that the significantly greater activation of CA1 neurons observed after the strong object memory training reflects the object exploration-induced encoding and consolidation of object memory rather than simply a consequence of exploring the familiar context.

Last, we utilized a naïve cohort of mice and qRT-PCR to quantify *Arc* mRNA expression in CA1 and PRh/LEC following strong and weak object memory training or in the respective yoked AH3 control groups, similar to that of our immunohistochemistry experiments. Analyses revealed no significant difference in *Arc* mRNA expression in the CA1 after strong object memory training compared to the strong AH3 control condition, and no significant difference in *Arc* mRNA expression in the PRh after weak object memory training compared to the weak AH3 control condition. Taken together with the results from the present inactivation and Arc protein expression studies, the Arc mRNA indicates a somewhat consistent pattern of results. We observed that strong object training, shown to require CA1 neuronal activity and perhaps CA1 synaptic plasticity, does not alter the net level of *Arc* mRNA in the CA1 relative to the strong AH3 condition. Likewise, weak object training, shown to require PRh neuronal activity and perhaps PRh synaptic plasticity, does not alter the net level of *Arc* mRNA in the PRh/LEC relative to the weak AH3 condition. In summary, the data suggest that object memory consolidation that promotes Arc protein expression does so without altering the level of *Arc* mRNA expression beyond that induced by the respective AH3 control condition. The pattern of results may also indicate that increases in Arc protein expression in the CA1 or PRh after strong or weak training, respectively deplete local stores of *Arc* mRNA, and homeostatic mechanisms are engaged to replenish basal levels of *Arc* mRNA.

A significant decrease in *Arc* mRNA in the PRh/LEC was observed after strong object memory training as compared to that after a strong AH3 control session (see Figure 4C). Note that Arc protein expression in PRh was significantly higher after weak object training compared to the weak AH3 condition. Collectively, the data suggest that if object exploration continues beyond the weak training, as it would during strong object training, then there is a significant increase in Arc protein expression in CA1, perhaps triggered by PRh dependent activation of hippocampal circuits consistent with the notion of transferal of object information from PRh to the hippocampus. The observed decrease in PRh/LEC *Arc* mRNA after strong training suggests two possibilities. One, the transfer of object information from PRh to the hippocampus requires significant translation (i.e., turnover) of available *Arc* mRNA in the PRh, which may account for the fact that Arc protein expression in PRh after strong object memory training is equivalent to that after a strong AH3 session. Thus, as strong object memory training disengages the PRh, *Arc* mRNA is down-regulated. Alternatively, the transfer from PRh to the hippocampus involves the active reduction of Arc protein built up during weak object training and the degradation of *Arc* mRNA in the PRh/LEC. This result alludes to the potential for a downregulating mechanism influencing baseline levels of *Arc* mRNA transcripts as a potential target underlying the recruitment of CA1 to support strong object memory (Bramham et al., 2008, 2010).

Analyses also revealed a decrease, albeit not significant, in *Arc* mRNA in the CA1 after weak object memory training compared to that after a weak AH3 control session (see Figure 4D). This result suggests that *Arc* mRNA expression is decreased in the brain region not engaged by the object memory training. Specifically, as weak object memory training did not engage the CA1 region beyond that of the weak AH3 session, *Arc* mRNA was degraded in the CA1. These results suggest that the increase in Arc protein observed in our immunohistochemical studies was not dependent on an increase in *Arc* mRNA expression. We acknowledge that the utilization of the biopsy punch for tissue sampling introduces variability, particularly in CA1 samples, which may include white matter and portions of the overlying cortex. A more precise tissue sampling technique (e.g., laser capture microscopy) may increase the likelihood of observing memory load-dependent differences in *Arc* mRNA expression in CA1. Together, the data suggest that weak object memory is dependent on the PRh/LEC and that there is a down-regulation of *Arc* mRNA in the PRh/LEC after strong memory training, which may promote the recruitment of CA1 activity to support the consolidation of strong object memory.

Collectively, these findings suggest that a sufficient degree of exploration of a novel or familiar object is necessary before synaptic plasticity is induced within the CA1 region, and thereafter CA1 neuronal activity is required to successfully consolidate the memory of the objects. Our previous work (Stackman et al., 2016) indicated that pre-test silencing of CA1 neuronal activity abolished test session novel object preference of mice that had completed strong object exploration criteria 24 h earlier. Therefore, it seems that the strong object memory-training event triggers synaptic plasticity within CA1, necessary for the consolidation (and subsequent retrieval) of the memory for the object exploration event, both of which are dependent on CA1 neuronal activity. It remains to be determined whether the pre-test inactivation of PRh neuronal activity affects novel object preference in mice that completed strong object exploration criteria. However, the present data suggest a transfer of critical regional neuronal activity supporting object memory consolidation from PRh to CA1, depending on the amount of time the mice are permitted to explore the novel objects during the sample session. We recently reported that muscimol inactivation of CA1 neuronal activity impaired novel object preference of strong memory trained mice when the test session was presented 20 min, but not 5 min, after the sample session (Ásgeirsdóttir et al., 2020). We interpreted that result as evidence that a working memory mechanism, perhaps in the PRh, supported object recognition when a <20 min delay was imposed between sample and test. Taken together with the present results, we propose that the transfer of object information from the PRh to the CA1 requires time, but also depends on a critical degree of memory load. These findings may be viewed as largely consistent with the notion that PRh is critical for object recognition based on familiarity; however, the recollection of the memory for the event of exploring novel objects within the spatial and temporal context of the testing arena requires CA1.

Furthermore, the immunohistochemical analyses of the brain tissue of mice that explored the novel objects for only 10 s each (i.e., the weak memory sample session group) indicate that the ensemble of Arc protein-expressing CA1 neurons was not significantly different from the CA1 ensemble in mice that explored the empty familiar arena (i.e., the weak memory AH3 group). Limiting the amount of novel object exploration during the sample session results in a weak memory, as demonstrated by significantly lower object discrimination exhibited during the test session. Our results reveal that the consolidation of such a weak object memory does not recruit CA1 neurons. Both AH3 mice and mice that received weak object training mice engaged in similar patterns of exploratory behavior and equivalent movement during their respective sessions; however, the information acquired is likely different, yet this difference was not reflected in CA1 Arc protein or mRNA expression. The lack of difference in CA1 Arc protein and mRNA expression could be interpreted in at least two ways.

One possibility is that the CA1 region may not contribute to the encoding of object memory, whether weak or strong. Testing the specific contribution of CA1 to object memory encoding will require silencing selective populations of neurons with high temporal precision. Certainly, pharmacological tools such as local muscimol microinfusion would not permit such highly precise time locking, although chemogenetic and optogenetic tools may be effective (Madisen et al., 2012; Boyden, 2015). The second possibility is that CA1 is only recruited after the mouse achieves some threshold amount of object exploration. Before reaching that object exploration threshold, the processing of memory for the objects is supported by PRh. This argument then suggests that there is some information threshold or storage buffer-like mechanism within PRh that once surpassed triggers the recruitment of CA1. A similar interpretation of mnemonic transfer from PRh to the hippocampus was stated in earlier reports (Gaffan and Parker, 1996; Liu and Bilkey, 1998), and more recent reports suggest that interactions between hippocampal regions and extra-hippocampal regions including the PRh are critical for encoding object-based episodic memory (Vilberg and Davachi, 2013). Such a transfer mechanism would be consistent with the downregulation of *Arc* mRNA observed in the PRh following strong object memory training compared to that observed in mice that experienced the AH3 control condition. Moreover, the significant increase in the count of Arc protein-positive PRh neurons of mice trained in the weak object memory protocol compared to the yoked AH3 group is consistent with the contribution of the PRh to object recognition, perhaps based on object familiarity. The finding that muscimol inactivation of PRh neurons impaired object memory encoded after limited exploration of objects, is also consistent with reports from studies of humans, nonhuman primates, and rodents, that damage to the PRh increases the rate of forgetting of recently acquired information (Wiig et al., 1996; Buffalo et al., 1998; Eichenbaum et al., 2007; Squire et al., 2007). These results are in agreement with previous findings that have shown that when mice explored sample objects for a limited amount of time (in this case 10 s on each object), test session object recognition was not impaired by hippocampal lesion or inactivation (Winters and Bussey, 2005; Winters et al., 2008). Taken together, these data imply that minimal exploration of novel objects promotes PRh neuronal activity; without recruiting CA1.

There is a limit to the interpretations one can draw from the results of traditional lesion studies related to the function of a specific brain region since the lesion technique renders the region of interest destroyed and unavailable to process incoming information. Temporary inactivation is an alternative technique that avoids some of the pitfalls of the lesion approach. Previous reports indicate that with a strong object memory protocol, pre-sample, or pre-test, intra-CA1 muscimol impairs object discrimination during the test session (De Lima et al., 2006; Cohen et al., 2013; Stackman et al., 2016; Ásgeirsdóttir et al., 2020). The results of the present regional inactivation studies extend the evidence demonstrating a significant role for CA1 in strong object memory. Additionally, the current immunohistochemical findings demonstrate that a significantly greater number of CA1 neurons are active after a strong sample session as compared to after a weak sample session, as represented by an increase in Arc expression. Also, the significant decrease in *Arc* mRNA expression in the PRh/LEC following a strong sample session suggests a down-regulation in that region. Alternatively, in a weak object memory protocol, the only inactivation of PRh led to impairments in test session object memory. These results are supported by the immunostaining findings showing increased neuronal activation in PRh following a weak object memory sample session. While infusion techniques have proven to be an effective treatment to temporarily inactivate a given region of the brain, the technique is limited (for review, see Cohen and Stackman, 2015). Future studies could employ optogenetics to selectively inactivate neurons in a given region for a discrete amount of time. This technique would allow for cellular inactivation only while the mice are exploring the objects to provide more precise control of the onset and offset of neuronal inactivation.

The present studies lend support to the notion that stronger event memories depend more upon the hippocampus than upon the PRh, as originally proposed by Squire et al. (2007). Their proposal was that the differences in memories supported by the hippocampus and PRh were determined by memory strength, suggesting that medial temporal lobe structures neighboring the hippocampus were responsible for encoding weak memory, as opposed to familiarity. Furthermore, the present studies provide molecular evidence for a phenomenon that has been well characterized on the behavioral level. Increased hippocampal activation, and down-regulation of *Arc* mRNA in PRh after the exploration of novel objects, and the observed impairments in object discrimination following CA1 inactivation in the strong object memory protocol, provides clear evidence for the contribution of the hippocampus to strong object recognition memory. On the other hand, in agreement with much of the literature on the effects of permanent lesions of PRh, the inactivation and immunohistochemical staining results after the weak object memory protocol add to the current knowledge about a functional role of PRh in the consolidation of a relatively weaker associative memory for objects.

This study provides evidence to suggest that both the PRh and the CA1 of the hippocampus in rodents support object memory processing, but that the recruitment of each structure depends on the strength of the memory, or the memory load, dictated by the amount of time the mouse engages in the exploration of the novel objects. Our present findings, which dissociate the contributions of the PRh and the CA1 region of the hippocampus to weak object memory and strong event memory, respectively, provide a unifying theory on object memory processing. Importantly, our results and the accompanying theory provide evidence for the necessity of both structures in object recognition memory.

## Data Availability Statement

The datasets generated for this study are available on request to the corresponding author.

## Ethics Statement

The animal study was reviewed and approved by the Institutional Animal Care and Use Committee of Florida Atlantic University.

## Author Contributions

DC designed and performed the experiments, analyzed data and wrote the article. SC designed and performed the experiments, analyzed data and wrote the article. KG designed the experiments. RS designed the experiments and wrote the article. All authors contributed to the article and approved the submitted version.

## Conflict of Interest

The authors declare that the research was conducted in the absence of any commercial or financial relationships that could be construed as a potential conflict of interest.
